# Effect of information on prostate biopsy history on biopsy outcomes in the era of MRI-targeted biopsies

**DOI:** 10.1007/s00345-020-03277-x

**Published:** 2020-05-29

**Authors:** Anna Lantz, Erik Skaaheim Haug, Wolfgang Picker, Alessio Crippa, Fredrik Jäderling, Ashkan Mortezavi, Tobias Nordström

**Affiliations:** 1 Department of Medical Epidemiology and Biostatistics, Karolinska Institutet, S-171 77 Stockholm, Sweden; 2 Department of Molecular Medicine and Surgery, Karolinska Institutet, Stockholm, Sweden; 3 Section of Urology, Vestfold Hospital Trust, Tønsberg, Norway; 4 Department of Radiology, Aleris Cancer Center, Oslo, Norway; 5 Department of Diagnostic Radiology, Karolinska University Hospital, Stockholm, Sweden; 6 Department of Clinical Sciences at Danderyd Hospital, Karolinska Institutet, Stockholm, Sweden

**Keywords:** Prostate cancer, Prostate neoplasm, Prostate biopsy, Targeted biopsy, Magnetic resonance imaging, MRI

## Abstract

**Purpose:**

To describe the predictive value of information on previous benign biopsy for the outcome of MRI-targeted biopsies.

**Methods:**

An exploratory analysis was conducted using data from a prospective, multicenter, paired diagnostic study of 532 men undergoing diagnostics for prostate cancer during 2016–2017. All men underwent 1.5 T MRI; systematic prostate biopsies; and MRI-targeted biopsies to MRI lesions with Prostate Imaging Reporting and Data System version 2, PI-RADS ≥ 3. The main outcome was numbers of detected prostate cancer characterized by grade group (GG) where GG ≥ 2 defined clinically significant cancer (csPCa).

**Results:**

Men with previous biopsies had significantly more often negative MRI (26% vs. 17%, *p* < 0.05) compared to men without previous biopsies. Men with previous biopsies showed higher rates of benign biopsies (41% vs. 26%, *p* < 0.05) and lower rates of GG2 (17% vs. 30%, *p* < 0.05) and GG ≥ 3 (5% vs. 10%, *p* < 0.05) cancer. Biopsy-naïve men had higher proportions of highly suspicious MRI lesions (PIRADS 5; *p* < 0.05) and a higher proportion of significant cancer in those lesions (*p* = 0.05). In multivariate regression analysis, a previous benign prostate biopsy was associated with less than half the odds of csPCa (OR 0.38; 95% CI 0.20–0.71).

**Conclusion:**

In this large prospective multicenter trial, we showed that men with a previous prostate biopsy had higher proportions of MRIs without lesions and lower proportion of highly suspicious lesions than biopsy-naïve men. Further, biopsy-naïve men showed higher detection of clinically significant cancer when using MRI-targeted biopsies. Also, in the era of MRI-targeted biopsy strategies, biopsy history should be carefully considered in biopsy decisions.

**Trial registration:**

NCT02788825 (ClinicalTrials.gov). Date of registration June 2, 2016.

**Electronic supplementary material:**

The online version of this article (10.1007/s00345-020-03277-x) contains supplementary material, which is available to authorized users.

## Introduction

Several studies have shown that MRI-targeted biopsy is superior to systematic transrectal ultrasonography-guided biopsy in men at clinical risk for prostate cancer [1–3]. For example, the PRECISION trial reported that prostate biopsies targeted to MRI-visible lesions detected more clinically significant cancer, less non-significant cancer and would safely spare some men with MRI not suggestive of prostate cancer from biopsy as compared with standard biopsies [1]. Recently, it was further demonstrated that a combined procedure combining targeted and systematic biopsies in men with visible MRI lesions enhances cancer detection and decreases risk of disease misclassification [4]. This supports the statement in EAU guidelines that this combined approach might be a suggested clinical standard [5].

Published studies however lack evidence whether this novel strategy for detection holds for both biopsy-naïve men and men with a previous prostate biopsy with a benign result. We recently published results from the paired design STHLM3MRI study where men without prior diagnosis of prostate cancer scheduled for prostate biopsy underwent both magnetic resonance imaging, systematic and two to three targeted fusion biopsies to any significant lesion defined as PIRADSv2 ≥ 3 at any of the three Scandinavian sites [6]. We found that a combination of a blood-based risk prediction tool and MRI-targeted biopsies can be used to inform biopsy decision making.

The aim of this analysis was to perform an exploratory analysis in the above cohort to evaluate how information on previous prostate biopsies affects MRI-targeted prostate biopsy outcomes to provide support for clinical decision making.

## Methods

This study describes an exploratory analysis using data from the previously published STHLM3 MRI Phase 1 study [6].

### Design

The STHLM3 MRI Phase study is a prospective, multicenter, paired diagnostic study registered as NCT02788825 (ClinicalTrials.gov). As previously described, men without prostate cancer, aged 45–75 years and referred for prostate biopsies or prebiopsy MRI, were recruited and underwent MRI and a combined prostate biopsy procedure. Any previous biopsy (yes/no) was reported by patients at study inclusion.

### MRI procedure

MRI was performed using a standardized, short, detection protocol compliant with European Society of Radiology Guidelines PI-RADS v2, except that dynamic contrast enhancement was omitted to decrease protocol complexity and acquisition time. We used 1.5 T magnetic field strength scanner at all study sites, without an endorectal coil. The protocol included T1- and T2-weighted images; diffusion-weighted images; *b*-values of 100, 450, and 800 with a calculated apparent diffusion coefficient map (ADC); and a separately acquired b1500. The time of acquisition was 16 min for the MRI sequences per participant.

MRI scans were reported according to the modified Prostate Imaging Reporting and Data System version 2 (following the PI-RADS v2 guidelines, with the modification that a PI-RADS 3 lesion in the peripheral zone may not be upgraded to PI-RADS 4 based on dynamic contrast) with up to three lesions with PI-RADS grade ≥ 3 marked for targeted biopsies. Any of six highly experienced uroradiologists reviewed the MRI series.

### Biopsy procedure

All participants underwent 10–12 systematic biopsies and patients with PI-RADS ≥ 3 underwent a combined biopsy procedure with two to three targeted biopsies to any marked lesion, performed by the same urologist. The systematic biopsies were sampled from the base, midpart and apex of the peripheral zone according to the Swedish National Guidelines for Prostate Cancer. The targeted biopsy procedure was performed before the systematic biopsies and by the same biopsying urologist. Targeted biopsies were undertaken using the Koelis system (Koelis Inc.), Artemis system (Eigen Inc.), or BioJet system (D&K Technologies GmbH). The systematic biopsies were performed without considering the location of the MRI lesions. The pathological specimen was locally reviewed by experienced uropathologists (SiV Tønsberg, Unilabs Stockholm, Oslo University Hospital). The biopsy cores were formalin fixed using separate containers and graded according to the International Society of Urological Pathology (ISUP) 2014 modification.

### Statistical methods

A PI-RADS score of ≥ 3 defined significant MRI lesions. The highest recorded ISUP Grade Group (GG) from MRI-targeted and systematic biopsies in men with significant MRI lesions defined the primary outcome. Secondary outcome was the GG of MRI-targeted biopsies alone. Clinically significant cancer was defined as ISUP grade group ≥ 2 cancer. We report the biopsy outcomes stratified by PI-RADS score (≤ 2, 3, 4, 5). Proportions were compared using the McNemar test where *p* < 0.05 was considered statistically significant. We modelled the primary outcome in a univariate and a multivariate logistic regression including biopsy status (any previous prostate biopsy; yes/no), age (years), PSA (ng/ml), PSA density (ng/ml^2^), PI-RADS score and pathological finding on digital rectal examination (DRE; yes/no) as predictors. We evaluated the receiver operating characteristics of the fitted logistic models calculating the area under the curve (AUC), compared using the DeLong method. All analyses were performed using Stata/MP 13.1 (Stata Corp., College Station, TX).

## Results

Baseline characteristics are presented in Table 1. 27% (*n* = 145) had a previous prostate biopsy with benign findings. In the overall cohort, the mean age was 58 years (SD 9) and median PSA density was 0.14 ng/ml^2^ [(IQR) 0.12], not statistically different between men with a previous biopsy and biopsy-naïve men. Men with a previous biopsy had significantly higher median PSA 8.0 vs. 6.0 ng/ml (*p* < 0.001), more pathological findings on digital rectal examination (DRE) 36% vs. 10% (*p* < 0.001), and less positive MRI findings (PI-RADS ≥ 3) 73% vs. 83% (P 0.014) compared to biopsy-naïve men.

**Table 1:** Participant characteristics

Variable	All (*n* = 532)	Biopsy naïve (*n* = 387)	Previous benign biopsy (*n* = 145)	p*
Age, year (mean, SD)	57.5 (9)	57.6 (9)	57.4 (9)	0.97
PSA, ng/ml (median, IQR)	6.3 (4.4)	6.0 (4.2)	8.0 (6.0)	< 0.001
PSA density, ng/ml^2^ (median, IQR)	0.14 (0.12)	0.13 (0.12)	0.14 (0.11)	0.10
Digital rectal examination				
Positive (%, *n*)	28.8 (148)	9.9 (14)	35.9 (134)	< 0.001
Negative (%, *n*)	71.2 (366)	90.1 (127)	64.1 (239)	
MRI lesion (PI-RADS ≥ 3)				
Yes (%, *n*)	80.6 (429)	83.2 (322)	73.4 (107)	0.014
No (%, *n*)	19.4 (103)	16.8 (65)	26.2 (38)	

*Comparing biopsy-naïve men and men with a previous benign biopsy

### Cancer detection

There was a higher proportion of men with cancer findings among biopsy-naïve men than in men with a previous biopsy (57% vs. 33%, *p* < 0.05). Specifically, the proportion of men with clinically significant cancer was higher in biopsy-naïve men (33% vs. 22%; *p* < 0.05; Table 2). Using systematic biopsies as end point, biopsy-naïve men showed a higher proportion GG1, GG2-3 and GG ≥ 4 findings, but lower proportion benign biopsy results. The median length of any cancer findings in systematic biopsies were longer in biopsy-naïve men than in previously biopsied men (10 mm vs. 5 mm; *p* < 0.05). The median length for any cancer finding on targeted biopsies was 15 mm (IQR 8–22) in biopsy-naïve men and 12 mm (IQR 4–22) in previously biopsied men (*p* = 0.2). Both when using systematic biopsies only and when omitting systematic biopsy results from the analysis, resembling the strategy of performing MRI-targeted biopsies only, we found a similar pattern (Table 2).

**Table 2:** Prostate biopsy findings by PSA naivety for different diagnostic strategies

	All (*n* = 532)	Biopsy naïve (*n* = 387)	Previous benign biopsy (*n* = 145)	p*
Using combined MRI-targeted biopsies				
Not performed, % (*n*)	19.4 (103)	16.8 (65)	26.2 (38)	0.02
Benign, % (*n*)	29.9 (159)	25.8 (100)	40.7 (59)	< 0.01
Grade Group 1, % (*n*)	15.6 (83)	17.3 (67)	11.0 (16)	0.07
Grade Group 2–3, % (*n*)	26.7 (142)	30.2 (117)	17.2 (25)	< 0.01
Grade Group ≥ 4, % (*n*)	8.5 (45)	9.8 (38)	4.8 (7)	0.07
Using MRI-targeted biopsies without addition of systematic biopsies				
Not performed	19.9 (106)	17.6 (68)	26.2 (38)	0.03
Benign, % (*n*)	34.2 (182)	30.8 (119)	43.5 (63)	0.02
Grade Group 1, % (*n*)	14.3 (76)	16.0 (62)	9.7 (14)	0.06
Grade Group 2–3, % (*n*)	24.3 (129)	27.4 (106)	15.9 (23)	< 0.01
Grade Group 4, % (*n*)	7.3 (39)	8.3 (32)	4.8 (7)	0.2
Using systematic biopsies				
Benign, % (*n*)	50.6 (269)	42.1 (163)	73.1 (106)	< 0.01
Grade Group 1, % (*n*)	19.0 (101)	21.2 (82)	13.1 (19)	0.03
Grade Grop 2–3, % (*n*)	24.1 (128)	28.2 (109)	13.1 (19)	< 0.01
Grade Group ≥ 4, % (*n*)	6.4 (34)	8.5 (33)	0.7 (1)	< 0.01

MRI-targeted biopsies were performed in men with significant MRI lesions defined as PI-RADS v2 ≥ 3. Results are displayed also when excluding results from systematic biopsies for men undergoing a targeted biopsy session

*Comparing biopsy-naïve men and men with a previous benign biopsy

### MRI results

19% (*n* = 103) of men showed no significant lesion on MRI. This proportion was lower among biopsy-naïve men [17% (*n* = 65)] than in men with a previous prostate biopsy, [26% (*n* = 38)] (*p* < 0.05); Table 3.

**Table 3:** MRI scorings and findings in corresponding target biopsy by biopsy naivety

	Biopsy naïve (*n* = 387)			Previous benign biopsy (*n* = 145)			*p*	*p*	*p*
	Total	csPCa findings	nsPCa findings	Total	csPCa findings	nsPCa findings	MRI	csPCa	nsPCa
PI-RADS score, % (*n*)	% (*n*)	% (*n*)	% (*n*)	& (*n*)	% (*n*)	% (*n*)			
≤ 2	16.8 (65)	–	–	26.1 (38)	–	–	< 0.05	–	–
3	36.2 (140)	16.4 (23)	20.7 (29)	37.9 (55)	12.7 (7)	1.8 (1)	0.7	0.5	< 0.05
4	23.5 (91)	44.0 (40)	26.3 (24)	20.7 (30)	30.0 (9)	30.0 (9)	0.5	0.2	0.7
5	23.5 (91)	82.4 (75)	9.9 (9)	15.2 (22)	63.6 (14)	18.2 (4)	< 0.05	0.05	0.3
Total	100 (387)	35.7 (138)	16 (62)	100 (145)	20.7 (30)	9.7 (14)			

Clinically significant prostate cancer (csPCa) was defined as ISUP grade group (GG) ≥ 2. Clinically non-significant cancer was defined as GG 1. Comparisons are described using *p* values from pairwise comparisons of proportions for MRI, csPCa and nsPCa findings between previously biopsied and biopsy-naïve men

Further analyzing the cancer detection by MRI scorings, Table 3 shows that biopsy-naïve men more frequently showed highly suspicious lesions on MRI (PIRADS 5; 23.5% vs. 15.2%, *p* < 0.05) and also showed higher detection of clinically significant cancer in those lesions than men with a previous biopsy (82.4% vs. 63.6%, *p* < 0.05). The negative predictive value of an MRI without significant lesions (PIRADS ≤ 2) was 92.3 (95% CI 83.0–97.5) in biopsy-naïve men, 94.7% (95% CI 82.3–99.4) among men with a previous biopsy and 93.2% (95% CI 86.5–97.2) for the whole study population.

We then performed a multivariate regression model including biopsy status (yes/no) to assess any independent association of biopsy status with the risk of clinically significant cancer. Illustrated in Table 4, men with a previous biopsy show significantly lower odds of significant prostate cancer (OR 0.38; 95% CI 0.20–0.71) independent of the other clinical parameters. When using systematic biopsies, a previous biopsy was independently associated with a lower risk of significant cancer (OR 0.25; 95% CI 0.13–0.49). Comparing AUC values for discrimination of csPCa, previous biopsy status added value when only PSA was considered. However, this was not conveyed in an increasing AUC when adding biopsy status to a full clinical model using age, digital rectal examination, PSA and PSA density (Table 5). The ROC curves are displayed in Supplementary Fig. 1.

**Table 4:** Uni- and multivariate logistic regression on risk of prostate cancer with ISUP Grade Group ≥ 2

	Univariate analysis			Multivariate analysis		
	OR	95% CI		OR	95% CI	
Using MRI-targeted biopsies						
Previous biopsy, years/*n*	0.47	0.30	0.74	0.38	0.20	0.71
Age, years	0.98	0.95	0.99	1.00	0.97	1.03
PSA, total	1.08	1.05	1.12	1.01	0.94	1.09
PSA density, ng/ml/cc (100)	1.07	1.05	1.09	1.04	1.01	1.07
PI-RADS score, ≤ 2–5	5.10	3.87	6.72	4.58	3.32	6.31
Digital rectal examination, pathological, 1/0	4.20	2.83	6.23	1.48	0.85	2.57
Using systematic biopsies						
Previous biopsy, years/*n*	0.28	0.17	0.46	0.25	0.13	0.49
Age, years	1.00	0.98	1.02	1.00	0.98	1.03
PSA, total	1.08	1.04	1.11	1.03	0.96	1.10
PSA density, ng/ml/cc (100)	1.06	1.04	1.08	1.06	1.03	1.09
Digital rectal examination, pathological, 1/0	5.26	3.52	7.86	4.32	2.71	6.90

**Table 5:** Discrimination for ISUP Grade Group ≥ 2 prostate cancer comparing logistic regression models using total PSA, data in previous biopsy and additional clinical information (age, PSA density)

	MRI-targeted biopsies with addition of systematic biopsies	MRI-targeted biopsies	Systematic biopsies
	AUC	AUC	AUC
Total PSA	0.60	0.62	0.61
Total PSA + Biopsy naivety	0.66	0.67	0.70
Clinical model	0.79	0.81	0.80
Clinical model + Biopsy naivety	0.79	0.80	0.81

Clinical model includes age, digital rectal examination, PSA, PSA-density

Finally, we assessed the added value of systematic biopsy in men with a significant lesion on MRI based on their biopsy status. Notably, omitting systematic biopsies in patients with a prior negative biopsy would have missed only two men (1.9%) with csPCa, but 18 (5.6%) in the biopsy-naïve cohort (*p* = 0.07).

## Discussion

In this exploratory analysis using data from a large prospective study of men undergoing MRI-targeted biopsies with or without the addition of systematic biopsies for prostate cancer detection, we demonstrate the added value of previous biopsy status for contemporary biopsy decisions. Our results show that men with a previous biopsy had significantly higher odds of MRI without significant lesions and lower odds of clinically significant prostate cancer, but no statistically significant difference in detection of non-significant prostate cancer. Correspondingly, biopsy-naïve men show higher odds of highly suspicious MRI lesions and also a higher frequency of significant cancer in such lesions. Adjusting for other clinical factors, we show that the odds of finding significant cancer in a man is more than halved if he has undergone a previous prostate biopsy.

Our results show that a history of a previous negative biopsy is strongly associated with lower risk of finding significant prostate cancer. Furthermore, our results show that systematic biopsies miss 20% of csPCa and that the added value of systematic biopsies in men with a previous biopsy are limited when performing MRI-targeted biopsies. In an analysis of 5519 men from the placebo group in the Prostate Cancer Prevention Trial (PCPT), results showed that history of a previous negative biopsy was associated with a decreased risk of overall prostate cancer (OR 0.64; 95% CI 0.53–0.78) and high-risk prostate cancer (OR 0.70; 95% CI 0.49–0.99) [7]. Furthermore, results from the European Randomized Study of Screening for Prostate Cancer (ERSPC) has shown that a previous negative biopsy reduces the likelihood of a biopsy-detectable high-grade prostate cancer (OR 0.32; 95% CI 0.17–0.61) [8]. However, both these studies were performed before the introduction of MRI in prostate cancer diagnostics.

Multiple studies have shown that MRI-targeted biopsies more accurately detect clinically significant PC while also detecting less indolent PC, compared to systematic transrectal ultrasonography-guided biopsies in men at clinical risk for prostate cancer [1, 2]. MRI and targeted biopsies have been extensively analyzed in the literature in distinction to different scenarios, as addition to or replacement for systematic biopsies, in the setting of a previous negative biopsy, as initial biopsy or during active surveillance. Regarding previous biopsy status, some studies have shown that MRI-targeted biopsies improve the detection rate in men with a previous biopsy compared to systematic biopsies, however, not in biopsy-naïve men [9, 10]. Furthermore, existing randomized controlled trials, performed in biopsy-naïve men, have reported conflicting evidence in the detection rate of clinically significant prostate cancer evaluating MRI with MRI-targeted biopsies vs. systematic biopsies [1, 11–13]. Thus, EAU guidelines recommend the use of MRI and targeted biopsies, in the setting of persistent clinical suspicion of prostate cancer in men with a previous negative biopsy. If the MRI is negative and clinical suspicion of prostate cancer remains, guidelines suggest performing systematic biopsy based on shared decision making with the patient.

Is there any evidence to support whether men with normal MRI need systematic biopsies if they had a prior benign biopsy? In a prospective multicenter randomized trial, including 665 men with a prior negative systematic biopsy and a persistent suspicion of prostate cancer (by either suspicious DRE or PSA > 4.0 ng/ml), results show that only 1.3% of clinically significant prostate cancers would have been missed if systematic biopsies had been omitted [14]. The authors conclude that the value of repeat systematic biopsies is limited in this setting.

Our results show when comparing AUC values for discrimination of clinically significant prostate cancer, previous biopsy status added value when only PSA was considered, however not when a clinical model including age PSA and PSA density was compared. Radtke et al. retrospectively analyzed 1015 men who underwent MRI prior to MRI/transrectal ultrasound fusion biopsy between 2012 and 2015. They added pre-biopsy MRI data to the ERSPC risk calculator to develop risk models for prediction of significant prostate cancer in biopsy-naive men (*n* = 660) and men after previous biopsy (*n* = 355). Their results also show that a previous negative biopsy was a significant predictor of clinically significant prostate cancer (*p* = 0.006). Furthermore, similar to our findings, their risk model, including MRI PI-RADS, PSA, age, prostate volume and DRE, reached a higher AUC at 0.83 for biopsy-naïve men, compared to an AUC of 0.81 in the same model for men with a previous biopsy [15]. Several studies have evaluated the combined systematic and targeted biopsies to assess the added value of each technique. These studies are merged in a recent Cochrane meta-analysis, including 43 studies, and results show that MRI and targeted biopsies are superior to systematic biopsies in making a correct diagnosis of clinically significant prostate cancer. In a stratified analysis based on previous biopsy status, using MRI-targeted biopsies, men with a previous negative biopsy were 44% more likely to make the correct diagnosis of clinically significant prostate, buts 5% in biopsy-naïve men, compared to systematic biopsies [3]. Thus, current evidence indicates that knowledge of previous biopsy history should be included in biopsy decisions and prediction models for clinically significant prostate cancer.

Our study has several strengths. First, this multi-center, prospective study using a paired design was specifically designed to study real-life performance of targeted biopsies. We use a structured and high-qualitative, short radiology protocol developed for early detection of prostate cancer and highly experienced uroradiologists to ensure high radiological quality. However, there are also obvious limitations. We present a pragmatic, exploratory analysis that was not pre-specified in the study protocol and conclusions are therefore primarily hypothesis generating. Second, we lack information on type and time span from the previous biopsy procedure which could impact the results. Third, the systematic biopsy was performed unblinded from the MRI results, possibly affecting the results of the systematic biopsies. Finally, although it has previously been shown that targeted biopsies decrease disease misclassification as compared with traditional biopsies [4], the true disease prevalence is unknown.

## Conclusion

In this large prospective multicenter study, we showed that men with a previous prostate biopsy had higher proportions of MRIs without lesions and lower proportion of highly suspicious lesions than biopsy-naïve men. Further, biopsy-naïve men showed higher detection of clinically significant cancer when using MRI-targeted biopsies and the added value of systematic biopsies in these men was limited. Also, in the era of MRI-targeted biopsy strategies, biopsy history should be carefully considered in biopsy decisions.

## Electronic supplementary material

Below is the link to the electronic supplementary material.

Supplementary Figure 1: Receiver Operating Characteristics curve comparing models including PSA and a clinical model with and without the addition of biopsy status (biopsy-naïve/previous biopsy) for predicting clinically significant cancer (ISUP ≥2) on combined MRI-targeted biopsy (DOCX 214 kb)

## Data Availability

This manuscript has associated data which will not be deposited.
