# Supplementary material for: Effect of information on prostate biopsy history on biopsy outcomes in the era of MRI-targeted biopsies
Source: World J Urol. 2020 May 29;39(4):1153–9. doi: 10.1007/s00345-020-03277-x (PMC8124045; doi:10.1007/s00345-020-03277-x)
Supplement: Supplementary file 1 — Supplementary Figure 1: Receiver Operating Characteristics curve comparing models including PSA and a clinical model with and without the addition of biopsy status (biopsy-naïve/previous biopsy) for predicting clinically significant cancer (ISUP ≥2) on combined MRI-targeted biopsy (DOCX 214 kb) [file 345_2020_3277_MOESM1_ESM.docx]

Supplementary Figure 1: Receiver Operating Characteristics curve comparing models including PSA and a clinical model with and without the addition of biopsy status (biopsy-naïve/previous biopsy) for predicting clinically significant cancer (ISUP ≥2) on combined

Supplementary Table 1: Prostate biopsy findings by PSA naivety for systematic biopsies performed in 532 men planned for biopsy or pre-biopsy MRI.

Supplementary Table 2: Uni- and multivariate logistic regression on risk of prostate cancer with Gleason Score ≥7.

Supplementary Figure 1: Receiver Operating Characteristics curve comparing models including PSA and a clinical model with and without the addition of biopsy status (biopsy-naïve/previous biopsy) for predicting clinically significant cancer (ISUP ≥2) on combined MRI-targeted biopsy.
